# Holistic Management of Pulmonary Fibrosis: A Comprehensive Review

**DOI:** 10.3390/medicina62050817

**Published:** 2026-04-24

**Authors:** Inés Palacio, Fernanda Hernández-González, Jacobo Sellarés, Jaume Bordas-Martinez

**Affiliations:** 1 Pulmonology Department, Hospital Clinic, 08402 Barcelona, Spain; ipalacio@clinic.cat (I.P.); fhernandez@clinic.cat (F.H.-G.); sellares@clinic.cat (J.S.); 2 Pulmonology Department, Hospital General de Granollers, 08402 Barcelona, Spain

**Keywords:** pulmonary fibrosis, interstitial lung disease, holistic care, multidisciplinary management, comorbidities, pulmonary rehabilitation, palliative care, psychosocial support, digital health, patient-centered care

## Abstract

Pulmonary fibrosis, encompassing idiopathic pulmonary fibrosis (IPF) and other fibrosing interstitial lung diseases (ILDs) with a progressive phenotype (PPF), represents a group of chronic, life-threatening conditions associated with significant morbidity, mortality, and socioeconomic burden. Despite advances in antifibrotic therapies, traditional disease-centered management alone is insufficient to address the multidimensional needs of affected patients. This comprehensive review advocates for a holistic, patient-centered approach to the management of pulmonary fibrosis, integrating pharmacological interventions with systematic comorbidity assessment, pulmonary rehabilitation, psychosocial support, nutritional optimization, early palliative care, social and community reinforcement, and digital health technologies. We examine the evidence supporting each dimension of holistic care, discuss current barriers to implementation—including healthcare fragmentation, limited multidisciplinary protocols, and scarce resources—and outline future perspectives centered on precision medicine and integrated care models. By shifting from a purely organ-focused paradigm to a comprehensive, multidisciplinary strategy, clinicians can improve not only disease outcomes but also quality of life and overall well-being for patients living with fibrosing ILDs.

## 1. Introduction

Interstitial lung diseases (ILDs) constitute a heterogeneous group of disorders characterized by varying degrees of inflammation and fibrosis of the lung parenchyma, small airways and alveoli [1]. ILDs encompass more than 200 entities historically grouped into broad clinical categories—idiopathic interstitial pneumonias (IIPs), connective tissue disease–associated ILDs, hypersensitivity pneumonitis, sarcoidosis, and ILDs related to specific occupational, environmental or drug exposures, alongside unclassifiable disease [2]. The 2025 ERS/ATS update of the multidisciplinary classification has substantially reframed this landscape by expanding the scheme beyond IIPs to incorporate secondary causes, refining nomenclature (with idiopathic diffuse alveolar damage replacing acute interstitial pneumonia, alveolar macrophage pneumonia replacing desquamative interstitial pneumonia, and bronchiolocentric interstitial pneumonia recognized as a major pattern), introducing an explicit fibrotic versus non-fibrotic subclassification of interstitial disorders, and incorporating diagnostic confidence into the framework [3]. Among ILD, idiopathic pulmonary fibrosis (IPF) represents the most common and severe form, with a median survival of 3–5 years from diagnosis [4]. The concept of progressive pulmonary fibrosis (PPF) has recently been formalized by the 2022 ATS/ERS/JRS/ALAT guideline as a clinical–behavioral construct describing non-IPF fibrosing ILDs that exhibit a progressive phenotype despite appropriate management of the underlying condition, sharing clinical behavior and prognosis similar to IPF [5]. The treatable traits framework has further moved practice toward management organized around individual patient characteristics rather than disease names [6]. This conceptual shift, from nosological classification toward behavior and trait-based stratification provides the rationale for a holistic management approach.

The pathogenesis of IPF reflects repeated alveolar epithelial micro-injury in genetically susceptible individuals, triggering aberrant epithelial–mesenchymal crosstalk, dysregulated fibroblast activation and myofibroblast differentiation, cellular senescence, and a self-perpetuating cycle of fibrotic remodeling that ultimately destroys lung architecture [7]. Genetic risk variants—including the *MUC5B* promoter polymorphism, telomere maintenance gene mutations, and surfactant protein variants—have advanced understanding of disease susceptibility and underscored impaired epithelial repair as a central pathobiological driver [7,8]. Several of the comorbidities that shape clinical course in IPF—pulmonary hypertension, gastroesophageal reflux disease, sleep-disordered breathing, lung cancer—appear mechanistically intertwined with these same pathways through shared aging-related, vascular and oxidative stress mechanisms [8], reinforcing the need for an integrated rather than organ-isolated management approach.

The global epidemiological burden of fibrosing ILDs is substantial and increasing. The prevalence of IPF ranges from 7 to 1650 per 100,000 people depending on the definition and geographic region, while the incidence of ILDs is difficult to specify owing to the large number of entities and heterogeneity in reported data [9]. An increasing trend in both incidence and prevalence has been described [10,11,12,13], largely attributable to improved disease recognition driven by greater clinician awareness, broader dissemination of updated diagnostic criteria, and expanded access to high-resolution imaging and other specialized diagnostic techniques. Furthermore, continuous therapeutic advances, along with increased availability of multidisciplinary expertise, have contributed to enhanced survival, thereby further increasing the number of individuals living with chronic fibrosing ILDs. These conditions impose a substantial healthcare and economic burden, with direct and indirect costs contributing meaningfully to national health expenditure across diverse healthcare systems [14,15,16,17].

Clinically, pulmonary fibrosis is characterized by relentlessly progressive restrictive ventilatory impairment, hypoxemia, exertional dyspnea, chronic cough, and reduced exercise tolerance, ultimately leading to respiratory failure [18,19,20]. However, the impact of these diseases extends far beyond pulmonary physiology. Patients with fibrosing ILDs frequently experience a high burden of comorbidities—cardiovascular disease, pulmonary hypertension, gastroesophageal reflux disease, sleep disorders, lung cancer, malnutrition, anxiety, and depression—all of which contribute to worsening symptoms, reduced quality of life, and increased mortality [21,22].

Despite advances in antifibrotic pharmacotherapy with nintedanib and pirfenidone, which slow the rate of lung function decline, these agents reduce the risk of acute exacerbations, and improve quality of life, but do not halt disease progression and do not address the complex, multidimensional needs of patients [23,24,25,26]. Lung transplantation remains the only intervention that improves survival, but it is only available in a minority of patients with advanced disease [5,27]. This therapeutic reality underscores the limitations of a purely disease-centered, organ-focused model of care.

**Figure 1:**
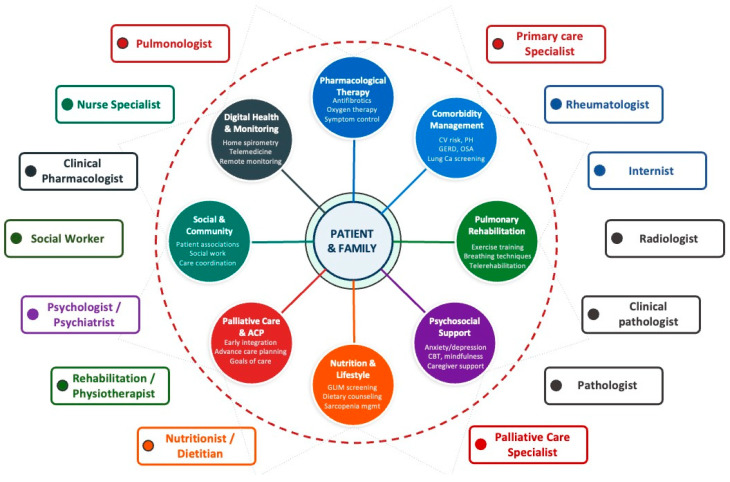
Patient-centered model for the holistic management of fibrosing interstitial lung diseases. The diagram illustrates the eight key domains of holistic care surrounding the patient, along with the multidisciplinary team members involved in coordinated management. ACP = Advance care planning; CBT = Cognitive–behavioral therapy; CV = Cardiovascular; GERD = Gastroesophageal reflux disease; GLIM = Global Leadership Initiative on Malnutrition; OSA = Obstructive sleep apnea; PH = Pulmonary hypertension; Ca = Cancer.

There is growing recognition that optimal management of pulmonary fibrosis requires a paradigm shift toward a holistic, patient-centered approach that systematically addresses not only the fibrotic lung disease itself but also the accompanying comorbidities, functional impairment, psychological distress, nutritional deterioration, social isolation, and existential suffering [28,29,30]. Such an approach demands coordinated multidisciplinary care encompassing diagnostic specialists (radiologists, pathologists), treating physicians (pulmonologists, rheumatologists, internists, primary care providers), allied health professionals (rehabilitation specialists, physiotherapists, psychologists, nutritionists, social workers), palliative care teams, and ILD specialist nurses [6,31,32] (Figure 1).

The aim of this comprehensive review is to provide a structured overview of the multiple dimensions of holistic care in pulmonary fibrosis, examine the evidence supporting each component, identify current barriers and challenges to implementation, and outline future perspectives for integrated care models and precision medicine approaches.

While narrative reviews on IPF management have been previously published, the present review offers a distinct contribution by providing the most comprehensive and up-to-date integration of all eight dimensions of holistic care within a single structured framework. It incorporates recent advances in antifibrotic therapy, pulmonary rehabilitation guidelines, palliative care, social and community context frameworks, and emerging artificial intelligence (AI)/radiomics tools, explicitly addressing barriers to implementation and future precision medicine perspectives that are absent from comparable prior work.

## 2. Dimensions of Holistic Care

The management of fibrosing ILDs should not be conceptualized as a series of isolated interventions targeting individual problems, but rather as a comprehensive strategy in which each therapeutic dimension is integrated into a unified care plan. The identification and management of comorbidities, functional limitations, psychological needs, and social factors should be addressed as interconnected components that collectively influence prognosis and patient quality of life. In the following subsections, we examine each dimension of holistic care in detail.

### 2.1. Comorbidity Assessment and Management

Patients with fibrosing ILDs may carry a substantial burden of comorbidities that significantly impact disease course, symptoms, functional status, and survival [21,22,33]. Systematic assessment and proactive management of these comorbidities is therefore a cornerstone of holistic care.

Comorbidities in fibrosing ILDs can be conceptually divided into two categories with distinct management implications. The first encompasses conditions that are pathogenetically linked to the fibrotic process or share common disease-driving mechanisms—including pulmonary hypertension, gastroesophageal reflux disease, sleep-disordered breathing, and lung cancer—where the relationship is bidirectional or mechanistically intertwined and targeted management may directly impact the fibrotic disease course [8]. The second category comprises comorbidities that are co-occurring but not specifically linked to fibrosis—such as cardiovascular disease, nutritional disorders, anxiety, and depression—which nonetheless carry significant prognostic and functional implications. This distinction should guide clinical prioritization: pathogenetically linked comorbidities warrant proactive screening and early intervention as part of routine ILD management, while co-occurring conditions should be systematically identified and addressed within a coordinated multidisciplinary framework [5,6,8].

#### 2.1.1. Comorbidities Impacting Pulmonary Fibrosis

Pulmonary hypertension (PH) is one of the most clinically significant comorbidities in fibrosing ILDs. Estimated PH prevalence in general ILD is around 35%, which increases in advanced fibrotic stages, being reported up to 86% at the time of lung transplantation [34,35]. PH is independently associated with reduced exercise capacity, increased oxygen requirements, and markedly worse survival [34,35,36]. The pathogenesis is multifactorial, involving hypoxic vasoconstriction, vascular remodeling, and destruction of the pulmonary capillary bed by fibrotic tissue. Screening with echocardiography should be considered particularly in patients with disproportionate dyspnea or degree of DLCO decline disproportionate to FVC impairment [34,35,36]. Right heart catheterization remains the diagnostic gold standard and is recommended to confirm suspected PH-ILD and guide therapy when non-invasive evaluation is inconclusive or treatment decisions are being considered [35]. Specific pharmacotherapy for PH-ILD remains limited, most agents approved for pulmonary arterial hypertension have demonstrated minimal efficacy or potential harm in PH-ILD [37]. The recent approval of inhaled treprostinil for PH associated with ILD represents an important advance [38]. Several Phase 3 trials of pulmonary vasodilators in PH-ILD are currently underway, and emerging evidence on antifibrotic effects of inhaled prostacyclin (see Section 2.2.1) suggests that the therapeutic landscape for this comorbidity may evolve substantially over the next few years.

Gastroesophageal reflux disease (GERD) is highly prevalent in IPF patients, with a prevalence that may exceed 60%, and rates can surpass 80% when assessed using esophageal pH monitoring [39,40,41,42]. Chronic microaspiration of gastric contents has been hypothesized to contribute to ongoing alveolar epithelial injury and fibrotic progression, although the causal relationship remains debated [43]. The causal relationship between GERD and IPF has been deeply evaluated through bidirectional Mendelian randomization but remains incompletely understood [44]. The ATS/ERS/JRS/ALAT 2022 guidelines issue a conditional recommendation against routine anti-acid medication for the treatment of IPF as a disease-modifying strategy, reflecting the very low quality of supporting evidence [5]. This recommendation does not preclude the use of anti-acid therapy for the management of symptomatic GERD on its own clinical merits. Careful assessment for both typical and atypical reflux symptoms, and consideration of pH monitoring in selected patients, is warranted.

In practical terms, IPF patients with symptomatic GERD should be managed according to standard gastroenterological practice, independently of the IPF diagnosis. In asymptomatic patients with objective evidence of reflux on pH monitoring or impedance testing, the 2022 guideline does not support empirical anti-acid therapy for disease-modifying purposes; nevertheless, the biological plausibility of the microaspiration hypothesis and the favorable safety profile of proton pump inhibitors have led some expert groups to consider empirical treatment in selected cases, a practice that remains controversial in the absence of supporting trial data [5,39,43].

Sleep-disordered breathing (SDB), encompasses several distinct conditions, with obstructive sleep apnea syndrome (OSAS) being the most prevalent. Its estimated prevalence in ILD approaches 70%, and appears slightly higher in IPF [45], whereas central sleep apnea is less common but still reported in 8–22% of patients [46,47]. Nocturnal hypoxemia is also highly prevalent—affecting around 37% of individuals—and may involve both sustained desaturation and intermittent hypoxemic events linked to apnea–hypopnea episodes [46,47,48,49,50]. SDB is frequently associated with fatigue, impaired cognitive performance, increased cardiovascular risk, pulmonary hypertension, and a worse prognosis [46,47,48,49,50]. Clinical presentation is often minimally symptomatic, and commonly used sleep-screening questionnaires show limited sensitivity, leading many authors to recommend systematic sleep evaluation in ILD patients [46,49,50,51]. Current evidence on CPAP therapy is mainly observational and, although it consistently demonstrates good tolerability and suggests a possible survival advantage, these findings still require confirmation in randomized controlled trials [52,53,54,55]. Nocturnal oxygen therapy has shown some physiological benefits, but its clinical relevance remains uncertain, and available evidence is still exploratory [55,56,57].

Lung cancer represents a life-threatening comorbidity in IPF, arising from the convergence of shared risk factors—most notably tobacco exposure—as well as overlapping pathogenic mechanisms at the genetic, molecular, and epithelial–mesenchymal levels [58,59]. Beyond these common drivers, the profibrotic and chronically injured microenvironment in IPF is itself thought to facilitate carcinogenesis. Epidemiological analyses show that individuals with IPF have an approximately 6.4-fold higher risk of developing lung cancer after adjustment for age, sex, and smoking history, with meta-analytic prevalence estimates around 13–14% [60]. Concomitant lung cancer has also been consistently associated with markedly worse prognosis [61]. Given this elevated risk profile, expert groups advise a low threshold for close imaging follow-up when pulmonary nodules are detected [62,63]. Early diagnosis remains central to management, as radical surgical resection may be considered in select early-stage cases despite the substantial risk of postoperative acute exacerbation [64,65]. When surgical treatment is not feasible, therapeutic options such as radiotherapy, chemotherapy, and immunotherapy must be considered with caution due to their well-recognized potential to precipitate fibrotic progression or acute exacerbations in patients with IPF [66].

#### 2.1.2. Other Comorbidities in the Comprehensive Approach

Cardiovascular diseases, including ischemic heart disease (IHD), heart failure (HF), and arrhythmias, are common in patients with fibrosing ILDs and represent important predictors of mortality [21,22]. Shared risk factors such as smoking, systemic inflammation, and endothelial dysfunction contribute to this association. Accumulating evidence indicates that cardiovascular disease is a recurrent and clinically relevant comorbidity in ILD and contributes meaningfully to adverse long-term outcomes, including increased mortality risk [33,67]. Beyond its direct impact, cardiovascular involvement may also exacerbate dyspnea, limit functional capacity, and complicate the interpretation of respiratory symptoms in daily practice. Therefore, cardiovascular risk assessment and optimization should be an integral part of the management plan, particularly within a holistic, multidisciplinary approach to ILD care.

Although current ILD-specific guidelines do not provide a structured cardiovascular screening protocol [5], a pragmatic approach in clinical practice may include: a baseline 12-lead ECG to detect arrhythmias (particularly atrial fibrillation); transthoracic echocardiography to evaluate for pulmonary hypertension and left ventricular dysfunction; and systematic identification and optimization of modifiable cardiovascular risk factors including smoking cessation and the management of hypertension, dyslipidemia, and diabetes. Follow-up cardiovascular assessment may be considered when clinically indicated [21,68].

Nutritional disorders, including malnutrition and sarcopenia, are increasingly recognized as prevalent and clinically meaningful in pulmonary fibrosis. Evidence indicates that these conditions affect a substantial proportion of patients and frequently arise irrespective of the degree of lung function impairment, highlighting that nutritional decline can occur even in earlier disease stages [69,70]. Clinical phenotyping also underscores the complexity of body-composition abnormalities, with both low body weight and obesity linked to functional decline, reduced exercise tolerance, poorer quality of life, decreased tolerability of pharmacological treatments, and worse overall prognosis [67,71,72]. Thus, early systematic nutritional screening and personalized interventions become essential pillars of holistic ILD management, helping to mitigate risks and support better overall patient prognosis.

Anxiety and depression are frequently observed and often underdiagnosed in ILD populations. A recent European survey found that 78% of ILD patients reported anxiety or depression, with many expressing fear of disease progression, social isolation, and frustration [73]. These psychological comorbidities are addressed in greater detail in Section 2.4. The coexistence of multiple comorbidities makes the patient clinically complex and highlights the need for systematic screening protocols and a multidisciplinary management approach rather than treating ILDs as isolated pulmonary conditions [6,21,22].

### 2.2. Pharmacological Interventions

Pharmacological treatment in fibrosing ILDs should be integrated into a global therapeutic plan that considers interactions with comorbidities, tolerance profiles, and the overall impact on patient functionality and quality of life.

#### 2.2.1. Disease-Modifying Pharmacological Treatments

The antifibrotic agents nintedanib and pirfenidone represent the pharmacological cornerstone for slowing disease progression in IPF [24,25,26]. The INBUILD trial [23] established nintedanib efficacy in non-IPF progressive fibrosing ILDs, leading to a conditional recommendation by the ATS/ERS 2022 guideline [5]. Evidence supporting pirfenidone in PPF is acknowledged as promising but remains more limited [5]. It is critical to emphasize that antifibrotics slow the rate of FVC decline but do not reverse established fibrosis, underscoring the need for complementary non-pharmacological strategies [6,27,31].

In ILDs associated with connective tissue diseases (CTD-ILD) or other immune-mediated conditions, immunosuppressive and immunomodulatory therapies remain the cornerstone of management. Mycophenolate mofetil (MMF) is widely used as a first-line induction agent, particularly in systemic sclerosis-associated ILD (SSc-ILD), while cyclophosphamide (CYC), azathioprine, and rituximab serve as alternatives or rescue therapies depending on the underlying autoimmune disorder and disease severity [27,74]. It is essential to recognize that when an underlying autoimmune or inflammatory condition drives the ILD, optimization of the disease-specific therapy should take priority, with antifibrotic agents viewed as complementary to attenuate fibrotic progression when standard management proves insufficient [5,27,74].

There is ongoing uncertainty regarding the optimal timing and combination of antifibrotic therapies with immunosuppressive regimens. The 2022 guidelines consider combination therapy biologically plausible and clinically reasonable in selected patients, particularly those with CTD-ILD who continue to progress despite appropriate immunosuppression [5]. Current and forthcoming clinical trials are expected to clarify optimal combination strategies.

The pharmacological landscape of fibrosing ILDs is evolving rapidly. Two recent Phase 3 trials have provided positive results: nerandomilast—an oral preferential phosphodiesterase 4B (PDE4B) inhibitor—significantly attenuated FVC decline at 52 weeks compared with placebo, both in patients with IPF and with PPF [75,76], representing the first novel oral mechanism of action to demonstrate efficacy across the IPF/PPF spectrum in over a decade. In parallel, inhaled treprostinil—initially approved for pulmonary hypertension associated with ILD on the basis of the INCREASE trial [38]—has subsequently shown a smaller decline in FVC and a reduction in clinical worsening events in patients with IPF in the Phase 3 TETON-2 trial, supporting an antifibrotic effect beyond its pulmonary vasodilatory action [77]. A parallel Phase 3 trial in PPF (TETON-PPF) is ongoing [78].

These recent successes contrast with several large Phase 3 trials in IPF that have failed to meet their primary endpoints in the same period, including ziritaxestat (an autotaxin inhibitor; ISABELA 1 and 2 [79]) and pamrevlumab (an anti-connective tissue growth factor monoclonal antibody; ZEPHYRUS-1 [80]). Additional investigational compounds in active Phase 2/3 development include the lysophosphatidic acid receptor 1 (LPA_1_) antagonist admilparant, currently evaluated in the Phase 3 ALOFT-IPF and ALOFT-PPF trials, alongside earlier-stage translational approaches targeting cellular senescence through senolytic therapy [81,82]. Together, these developments illustrate both the growing therapeutic armamentarium and the methodological challenges of drug development in pulmonary fibrosis, where mechanistically promising compounds frequently fail to translate into clinically meaningful efficacy.

Lung transplantation remains the only intervention with the potential to substantially alter the disease trajectory in patients with severe or rapidly progressive fibrosing ILD who do not respond to medical therapy and have no contraindications. Early referral for transplant evaluation is recommended, given the unpredictable disease course and waiting list constraints [27,83]. Patients with telomere-related forms of fibrosing ILD constitute a growing subgroup referred for lung transplantation, often at younger ages and with faster disease progression. Systematic review of available literature has shown that patient and graft survival after lung transplantation in telomere-related fibrosing ILD do not appear inferior to those of patients without telomere dysfunction, although specific post-transplant management considerations may apply [84].

#### 2.2.2. Pharmacological Treatments for Symptom Control

Supplemental oxygen therapy is widely used to manage hypoxemia in fibrosing ILDs. Although ILD-specific evidence remains limited, and many recommendations are extrapolated from COPD, current practice supports long-term oxygen therapy (LTOT) for patients with severe resting hypoxemia and ambulatory oxygen for those with significant exertional desaturation, primarily to improve exercise capacity and health-related quality of life [85,86,87,88]. Oxygen therapy should be prescribed through an individualized, patient-centered approach that balances symptomatic benefit with treatment burden, mobility restrictions, and psychosocial impact [89]. Nevertheless, the clinical relevance of its impact on daily life remains under discussion [57,90,91].

Dyspnea is the cardinal and most distressing symptom, profoundly affecting quality of life. The European Respiratory Society (ERS) recommends a stepwise strategy combining non-pharmacological and pharmacological interventions [5,92]. Despite limited high-quality evidence, emerging data and clinical experience support the judicious use of low-dose opioids for refractory dyspnea when other measures have been optimized, although the optimal timing for initiation remains uncertain and treatment decisions should be individualized [93,94,95].

Chronic cough in fibrosing ILDs is multifactorial, with contributors including ongoing fibrotic activity, rhinitis, GERD, medication effects (e.g., pirfenidone), and neural cough hypersensitivity. Management should prioritize identification and treatment of modifiable causes. Opioids and neuromodulators (e.g., gabapentin, pregabalin) may be considered for refractory cases, while speech therapy and pulmonary rehabilitation programs can offer additional benefit [93,95,96,97,98].

### 2.3. Pulmonary Rehabilitation

Pulmonary rehabilitation (PR) is a comprehensive intervention that encompasses exercise training, health education, and behavioral change, designed to improve the physical and psychological condition of patients with chronic respiratory diseases. A Cochrane systematic review confirmed that PR improves exercise capacity (6 min walk distance), reduces dyspnea, and enhances health-related quality of life in patients with ILDs, with benefits observed across various disease subtypes [99].

The 2023 American Thoracic Society (ATS) clinical practice guideline provides a strong recommendation for pulmonary rehabilitation in adults with chronic respiratory disease, including ILDs, emphasizing its role as an essential component of comprehensive management [100]. PR programs for ILD patients should be tailored to individual functional capacity, disease severity, and the presence of comorbidities, particularly cardiovascular and musculoskeletal conditions, that may limit exercise tolerance and require modified training protocols [101].

Beyond physical conditioning, PR serves as a platform for patient education regarding disease self-management, energy conservation techniques, and emotional support through peer interactions and group dynamics. It provides an opportunity for empowerment and emotional adaptation, helping patients develop coping strategies and maintain an active role in their care. Key components include supervised aerobic and resistance exercise training, breathing techniques (including pursed-lip breathing and diaphragmatic breathing), education on oxygen use and medication management, and psychosocial counseling [99,100,101].

Despite the established benefits of PR, access remains limited for many ILD patients due to geographic barriers, insufficient program availability, and resource constraints. Home-based and telerehabilitation programs are emerging as viable alternatives to center-based models, potentially expanding access while maintaining clinical effectiveness [102,103]. Research is ongoing to define optimal program duration, intensity, maintenance strategies, and the role of technology-supported delivery models.

### 2.4. Psychosocial Support and Mental Health

The psychological burden of fibrosing ILDs is substantial and often underrecognized. A recent cross-sectional online survey of 59 ILD patients in Western Europe (77% on antifibrotic therapy) reported that 78% experienced anxiety or depression, frequently related to fears about disease progression, social isolation, and loss of autonomy. Physical activity was also markedly reduced, with 55% being mobile for less than two hours per day, 73% reporting mobility limitations, and 51% requiring oxygen during exertion. Only around 20% reported no emotional impact from their condition [73].

Psychological support should be integrated into the therapeutic plan from early disease stages, particularly in patients with pre-existing psychiatric disorders or significant functional impairment. High rates of anxiety and depression have been consistently documented across ILD populations, and these conditions are independently associated with worse quality of life, reduced treatment adherence, and poorer clinical outcomes [73,104,105,106,107,108].

There is a notable scarcity of psychological data focused specifically on fibrosing ILD, with most current findings extrapolated from heterogeneous ILD populations or from non-ILD chronic respiratory disease cohorts. Given this limitation, current clinical practice should adopt a pragmatic approach: routine screening with validated, brief instruments—such as the Hospital Anxiety and Depression Scale [109], the Patient Health Questionnaire-9 [110] and the Generalized Anxiety Disorder-7 scale [111]—at diagnosis and at major clinical transitions (such as functional decline, transplant evaluation, or transition to palliative care), with referral to psychological support when validated thresholds are exceeded.

Regarding psychological and behavioral interventions in ILD, the evidence is even more limited, with only a few interventional studies conducted to date. Available data suggest that approaches such as mindfulness-based stress reduction, acceptance and commitment therapy, support groups, and caregiver-focused programs may offer benefit, but robust trial evidence is scarce. In a randomized trial involving predominantly non-ILD patients with chronic respiratory disease (<5% ILD), Cognitive–behavioral therapy (CBT) led to improvements in depressive symptoms over time, although no significant differences in anxiety or depression were observed compared with pulmonary rehabilitation alone [112]. More encouraging findings come from disease-specific studies: an online mindfulness-based cognitive therapy program in sarcoidosis improved fatigue, anxiety, depressive symptoms, and overall health [113], and a 9-week digital CBT intervention in patients with IPF demonstrated meaningful reductions in anxiety symptoms [114].

Psychological distress also extends to caregivers, who frequently experience high levels of burden, uncertainty, and emotional strain. Addressing caregiver needs, coping resources, and support structures should therefore form part of a comprehensive ILD care model [28,29].

Finally, routine screening for anxiety, depression, and psychological distress using validated tools should be implemented at diagnosis and periodically throughout the disease course to facilitate timely identification and management [6,21,22].

### 2.5. Nutrition and Lifestyle

Nutritional status is increasingly recognized as a significant determinant of outcomes in fibrosing ILDs. The lungs serve not only in gas exchange but also as structural and immunological barriers, and their function is influenced by systemic metabolic and nutritional factors [115]. Deficiencies in vitamins A, C, and D, as well as inadequate intake of omega-3 fatty acids, have been associated with impaired lung barrier function, altered immune homeostasis, and enhanced inflammatory signaling, increasing vulnerability to ongoing lung injury [115].

Malnutrition and sarcopenia are highly prevalent in pulmonary fibrosis patients and may significantly affect disease trajectory and functional capacity. The study by Cabrera-César et al. demonstrated that 77.6% of IPF patients met Global Leadership Initiative on Malnutrition (GLIM) criteria for malnutrition, with 20% also exhibiting sarcopenia. The finding that nutritional deterioration occurred independently of the degree of lung function impairment suggests that nutritional screening should be performed routinely, regardless of disease stage [69].

Lifestyle factors beyond nutrition also warrant attention. Physical inactivity, obesity, and active smoking promote chronic low-grade systemic inflammation and metabolic dysfunction, which may accelerate fibrotic processes [115,116]. Environmental pollutants, including air pollution, particulate matter, occupational exposures, and climate-related pollutants, induce oxidative stress, inflammation, and epithelial barrier damage in the lungs [117,118]. Antigen avoidance is particularly critical for patients with hypersensitivity pneumonitis [119].

Although routine nutritional screening using GLIM criteria is increasingly supported by the high prevalence of malnutrition in this population, robust interventional trial evidence for specific nutritional strategies in fibrosing ILD remains very limited. Nutritional intervention should therefore be adapted to the patient’s physical, metabolic, and social limitations as part of the holistic approach, integrating individualized dietary counseling, oral nutritional supplementation when indicated, and exercise-based strategies to combat sarcopenia within the multidisciplinary care plan [69,70,101]. Vaccinations and prophylactic measures to prevent respiratory infections complement these lifestyle recommendations [120].

### 2.6. Palliative Care and Advance Care Planning

Palliative care (PC) in fibrosing ILDs should be understood as an integral component of active management rather than an intervention reserved for the terminal phase. The 2023 ERS clinical practice guideline defines palliative care for people with COPD or ILD as “a holistic and multidisciplinary person-centered approach aiming to control symptoms and improve quality of life of people with serious health-related suffering, and to support their informal caregivers” [92]. The guideline recommends considering palliative care when patients and their caregivers have physical, psychological, social, or spiritual/existential unmet needs, emphasizing that this assessment should occur early and be revisited regularly throughout the disease course.

The three most common and distressing symptoms in fibrosing ILDs, dyspnea, cough, and fatigue, often persist despite optimal disease-directed therapies and require a structured, symptom-focused approach. Dyspnea is best addressed through a multimodal strategy that combines progression-slowing effects of antifibrotics with non-pharmacological measures such as exercise training, breathing techniques, and CBT for anxiety-related breathlessness, while low-dose opioids remain a last-line option for refractory symptoms. Effective cough control depends on identifying and treating dominant contributors such as rhinitis, reflux, medication effects, or neural hypersensitivity; neuromodulators or low-dose opioids may be used in refractory cases, and speech therapy can provide additional benefit. Fatigue management begins with targeted evaluation of reversible factors, including sleep disorders, mood disturbances, medication effects, and deconditioning, followed by individualized rehabilitation and supportive interventions [6,31,94,96].

Advance care planning (ACP) is a fundamental component of palliative care in fibrosing ILDs, a priority emphasized in a recent expert call to action [121]. Given the unpredictable disease course and risk of acute exacerbations with high short-term mortality, early and iterative discussions about goals of care, treatment preferences, and end-of-life wishes are essential. A retrospective study by Chai et al. found that ILD patients who received outpatient palliative care had higher uptake of ACP (39% vs. 11%), increased opioid prescribing for symptom relief (96% vs. 27%), and lower frequency of intensive care unit admissions (5% vs. 19%) compared with usual care, with a longer median survival of 23.9 months vs. 11.4 months [122]. Empathetic communication and shared decision-making should guide all discussions regarding the goals and limitations of treatment.

### 2.7. Social and Community Support

The social environment can act as a powerful facilitator or barrier to disease management and patient perception. Patients with fibrosing ILDs experience significant restrictions in physical functioning and psychosocial well-being, and their lived experience is shaped not only by medical treatment but also by the quality of their social support networks [123,124].

Family members and caregivers play a critical role in supporting patients with daily activities, treatment management, and emotional coping. Support from close relatives helps patients adapt to the physical limitations of the disease and maintain engagement with treatment and healthcare services. However, the caregiving role itself carries significant physical and emotional burden, and caregiver well-being should be actively assessed and supported as part of comprehensive care [28,125,126].

Patient associations and peer support groups provide valuable platforms for shared experiences, practical advice, and emotional solidarity. Engagement with these communities has been identified as an important facilitator of emotional well-being and disease management in ILD surveys [127,128]. Coordination between levels of care, from tertiary ILD centers to primary care providers and community-based services, is essential to ensure continuity and accessibility of support across the disease trajectory [123,125,129].

Impact of family and social environment on treatment adherence and well-being should be routinely assessed. Social workers, when available, can help identify unmet social needs, facilitate access to financial resources and disability benefits, and coordinate community-based support services [130]. Strengthening these social support structures is an integral but often overlooked dimension of holistic ILD care.

### 2.8. Technology and Digital Health

Digital health technologies are increasingly being integrated into the management of fibrosing ILDs, enabling more continuous, personalized, and patient-centered care. Remote monitoring systems, including home spirometry, pulse oximetry, and smartphone-based symptom tracking, allow clinicians to assess disease progression and treatment response outside traditional clinical settings, potentially enabling earlier detection of clinical deterioration [131,132]. The real-world feasibility and reliability of these tools is being progressively established. For example, large-scale nationwide data have demonstrated sustained adherence to home spirometry over one year, while ILD-specific smartphone applications show high usability and consistent engagement, particularly for physical activity tracking and environmental monitoring [133,134]. Although numerous telehealth studies in ILD populations have been conducted, the evidence base remains methodologically heterogeneous, with most studies being feasibility or single-center observational cohorts vulnerable to selection and publication bias, and Phase 3 randomized controlled trials demonstrating clinical-outcome benefit (rather than process measures) are still ongoing [135,136].

A recent narrative review on remote patient monitoring in autoimmune-related ILD highlighted that device-derived signals and patient-generated health data show useful agreement with clinic measures when interpreted across repeated time points, supporting rehabilitation, self-management, and timely clinical decision-making [137]. Despite this potential, important limitations persist, including data variability, interoperability issues, workforce implications, and inequities in digital access.

Telemedicine further facilitates remote consultations, reduces travel burden for patients with limited mobility, and enables more frequent clinical contact. Even minimal-infrastructure communication channels, such as offering patients a direct telephone or email contact, have been associated with improvements in patient experience [138], reinforcing that meaningful digital support does not always require expensive or sophisticated technological deployment. Digital tools for patient education, self-management support, and care coordination hold substantial promise, but successful implementation requires attention to digital literacy, equitable access, data privacy, and integration with existing electronic health records [131,137].

Radiomics and AI are emerging as transformative tools in the management of fibrosing ILDs. Radiomic feature extraction from HRCT enables quantitative characterization of fibrosis extent and spatial heterogeneity beyond visual assessment, with demonstrated prognostic value in IPF. Deep learning algorithms applied to HRCT have shown performance comparable to expert radiologists in ILD pattern recognition tasks and are being validated in multicenter real-world settings. AI-based automated fibrosis quantification offers objective, reproducible longitudinal tracking of disease extent, potentially enabling earlier detection of progression than conventional pulmonary function test thresholds. Integration of radiomic data with clinical, functional, and molecular variables through multimodal machine learning models is being explored for patient phenotyping, risk stratification, and personalized treatment selection [139,140,141,142]. However, challenges remain: most models lack prospective validation in independent cohorts, training datasets are often small and single-center, and concerns persist regarding reproducibility across different CT acquisition protocols. Equitable access, regulatory approval pathways, and clinical integration into existing workflows represent further barriers to routine implementation.

## 3. Barriers and Challenges

Despite the compelling rationale for holistic management, several significant barriers impede its systematic implementation in clinical practice.

Fragmentation of healthcare systems represents perhaps the most fundamental obstacle. Current healthcare models typically organize care around diagnostic labels rather than patient-specific characteristics, leading to compartmentalized management in which pulmonary complications, systemic diseases, comorbidities, and lifestyle factors are addressed separately across different specialties. Without coordinated multidisciplinary care, patients face delayed diagnosis, inconsistent treatment strategies, poor inter-specialty coordination, and suboptimal management of comorbidities. The treatable traits framework proposed by Khor et al. offers a promising conceptual approach to restructuring care around individual patient characteristics rather than disease labels, but its practical implementation remains challenging [6].

Lack of integrated protocols for multimorbidity management is a related challenge. While clinical practice guidelines for fibrosing ILDs have evolved substantially [5], they remain primarily focused on pharmacological management and often do not provide comprehensive frameworks for integrating comorbidity screening, rehabilitation, psychological support, and palliative care into routine pathways. Development and implementation of structured, sequential diagnostic and therapeutic approaches could help bridge this gap. Qualitative studies exploring patient, caregiver, and clinician perspectives increasingly highlight the need to establish a structured patient journey for the diagnosis and multidisciplinary management of fibrosing ILDs, underscoring gaps in coordination, communication, and continuity of care [73,129,143,144].

Limited resources and the need for specialized training pose additional barriers. Fibrosing ILDs are uncommon conditions, and many healthcare professionals outside specialized centers receive limited training in their recognition and management. This leads to delayed or incorrect diagnoses, particularly for rarer ILD subtypes. Access to specialized diagnostic tools (e.g., high-resolution CT, surgical lung biopsy, multidisciplinary discussion) and multidisciplinary expertise is often restricted, contributing to inequalities in patient care. Improved clinician training, increased awareness, and stronger referral networks to specialized centers are essential to ensure timely and appropriate management. Furthermore, the geographic and logistical barriers to pulmonary rehabilitation access, the limited availability of psychosocial services, workforce constraints in palliative care, and the nascent state of digital health infrastructure all contribute to a gap between the ideal of holistic care and its practical delivery [145,146,147,148].

## 4. Future Perspectives

Development of multidisciplinary teams (MDTs) remains an important organizational challenge to deliver holistic ILD care. Expanding the diagnostic multidisciplinary teams to include rehabilitation professionals, palliative care specialists, psychologists, nutritionists, social workers and primary care would enable comprehensive care planning and monitoring [32,149]. The COCOS-IPF project’s European survey on healthcare professionals’ views highlighted that person-centered health outcomes are increasingly recognized as important but are inconsistently implemented in routine clinical practice [150,151].

Precision medicine and biomarker-guided therapy represent the next frontier in personalized ILD management. Traditional “one-size-fits-all” treatment approaches do not adequately account for the variability in disease course or treatment response across patients. Advances in identifying molecular endotypes, genetic susceptibility markers, and circulating biomarkers are enabling risk stratification, prognostication, and targeted therapy selection based on individual clinical profiles rather than disease labels alone [152,153,154]. Recent positive trials for nerandomilast and inhaled treprostinil are expanding the antifibrotic armamentarium beyond pirfenidone and nintedanib, reinforcing the urgency of precision-based approaches to individualized treatment selection [75,76,77]. However, translating both these molecular insights and emerging therapies into routine clinical practice faces significant infrastructure, regulatory, financial, and ethical barriers.

Research on non-pharmacological interventions and integrated care models is urgently needed. While the evidence base for pulmonary rehabilitation in ILD continues to grow, there is a critical need for well-designed trials evaluating the effectiveness of psychosocial interventions, nutritional strategies, digital health tools, and integrated palliative care pathways specifically in fibrosing ILD populations [6,28,150,155]. Defining core outcome sets that capture the multidimensional impact of these conditions, including patient-reported outcomes, caregiver burden, and health-economic endpoints, will be essential for evaluating the effectiveness of holistic care models.

Additionally, the integration of artificial intelligence and machine learning into ILD care, for automated imaging analysis, outcome prediction, and treatment optimization, holds promise for enhancing diagnostic accuracy and personalizing management strategies [131,132]. Table 1 summarizes the main current gaps and future perspectives in holistic ILD management.

## 5. Conclusions

IPF and other fibrosing ILD are progressive conditions whose impact extends far beyond pulmonary physiology. The clinical course and patient well-being are profoundly influenced by comorbidities, refractory symptoms, psychological distress, nutritional deterioration, and social factors, all of which contribute to reduced quality of life and increased healthcare utilization. These characteristics underscore the limitations of traditional disease-centered models and highlight the urgent need for a comprehensive, patient-centered approach to care.

Optimal management requires the integration of evidence-based pharmacological therapies with systematic comorbidity identification and treatment, structured pulmonary rehabilitation, psychological and behavioral support, nutritional assessment and intervention, early palliative care, social reinforcement, and digital health technologies. Comprehensively addressing these dimensions allows clinicians not only to slow disease progression but also to alleviate symptoms, preserve functional capacity, and improve patients’ overall well-being. Multidisciplinary collaboration among pulmonologists, rheumatologists, palliative care specialists, rehabilitation professionals, psychologists, nutritionists, social workers, and primary care providers is essential to deliver individualized and continuous care.

Advances in digital health technologies, remote monitoring, and precision medicine are creating new opportunities for more proactive and personalized disease management. However, important challenges remain, including fragmented healthcare pathways, delayed diagnosis, limited access to specialized expertise, and insufficient integration of multimorbidity management within existing care models. Strengthening multidisciplinary care pathways, improving access to specialized services, investing in clinician education, and further developing integrated models of care will be critical to improving clinical outcomes and quality of life for patients living with fibrosing interstitial lung diseases.

## Figures and Tables

**Table 1:** Current gaps and future perspectives in holistic management of fibrosing interstitial lung diseases.

Domain	Current Gap/Barrier	Future Perspective
Healthcare organization	Fragmented care organized around diagnostic labels rather than patient-specific traits	Expanded MDTs; implementation of the treatable traits framework
Clinical guidelines	Focused on pharmacology; lack integrated protocols for multimorbidity	Structured patient journey; multimorbidity care pathways
Workforce and training	Limited ILD expertise outside specialized centers; delayed or incorrect diagnoses	Clinician education programs; stronger referral networks to specialized centers
Pulmonary rehabilitation	Geographic barriers; limited program availability and resource constraints	Home-based and telerehabilitation models; technology-supported delivery
Psychosocial support	Limited evidence base; insufficient mental health services in ILD settings	Disease-specific CBT and mindfulness trials; routine psychological screening
Nutrition	Routine nutritional screening not implemented; high prevalence of malnutrition unrecognized	Integrated nutritional assessment; anti-sarcopenia strategies within rehabilitation
Palliative care	Often reserved for terminal phase; workforce constraints; insufficient advance care planning	Early integration from diagnosis; ACP as standard practice; symptom-focused protocols
Digital health	Data variability; interoperability issues; digital access inequities	Standardized remote monitoring; AI-driven tools; equitable digital infrastructure
Precision medicine	One-size-fits-all treatment approaches; limited biomarker translation to clinic	Biomarker-guided therapy; molecular endotyping; risk stratification; individualized antifibrotic selection
Research	Insufficient trials on non-pharmacological interventions; lack of core outcome sets	Well-designed trials for psychosocial, nutritional, and digital interventions; patient-reported outcome sets

## Data Availability

No new data were created or analyzed in this study.
